# Integrative analyses of targeted metabolome and transcriptome of Isatidis Radix autotetraploids highlighted key polyploidization-responsive regulators

**DOI:** 10.1186/s12864-021-07980-w

**Published:** 2021-09-17

**Authors:** Zixuan Zhang, Mingpu Tan, Yingying Zhang, Yue Jia, Shuxian Zhu, Jiang Wang, Jiajing Zhao, Yueyue Liao, Zengxu Xiang

**Affiliations:** 1 College of Horticulture, Nanjing Agricultural University, 210095 Nanjing, China; 2 College of Life Sciences, Nanjing Agricultural University, 210095 Nanjing, China

**Keywords:** Polyploidization, Metabolome, Transcriptional factors, lignan biosynthesis, Isatidis Radix

## Abstract

**Background:**

Isatidis Radix, the root of *Isatis indigotica* Fort. (Chinese woad) can produce a variety of efficacious compound with medicinal properties. The tetraploid *I. indigotica* plants exhibit superior phenotypic traits, such as greater yield, higher bioactive compounds accumulation and enhanced stress tolerance. In this study, a comparative transcriptomic and metabolomic study on Isatidis Radix autotetraploid and its progenitor was performed.

**Results:**

Through the targeted metabolic profiling, 283 metabolites were identified in Isatidis Radix, and 70 polyploidization-altered metabolites were obtained. Moreover, the production of lignans was significantly increased post polyploidization, which implied that polyploidization-modulated changes in lignan biosynthesis. Regarding the transcriptomic shift, 2065 differentially expressed genes (DEGs) were identified as being polyploidy-responsive genes, and the polyploidization-altered DEGs were enriched in phenylpropanoid biosynthesis and plant hormone signal transduction. The further integrative analysis of polyploidy-responsive metabolome and transcriptome showed that 1584 DEGs were highly correlated with the 70 polyploidization-altered metabolites, and the transcriptional factors TFs-lignans network highlighted 10 polyploidy-altered TFs and 17 fluctuated phenylpropanoid pathway compounds.

**Conclusions:**

These results collectively indicated that polyploidization contributed to the high content of active compounds in autotetraploid roots, and the gene–lignan pathway network analysis highlighted polyploidy–responsive key functional genes and regulators.

**Supplementary Information:**

The online version contains supplementary material available at 10.1186/s12864-021-07980-w.

## Introduction

*Isatis indigotica* Fort. (Chinese woad, 2*n* = 14) belongs to *Isatideae* tribe of the *Brassicaceae* family. The root of *I. indigotica* called Isatidis Radix, which can produce a variety of chemicals with medicinal properties, can be used in clinical treatment of regular seasonal influenza and plays an immune regulatory role in vitro and in vivo [1], while the leaves of *I. indigotica* called Isatidis Folium composed of isatin, tryptanthrin, indirubin and so on [2, 3]. Several categories of metabolites including alkaloids, phenylpropanol, organic acids and polysaccharides identified from Isatidis Radix, were demonstrated to achieve the antiviral and antioxidant effects [4–6]. Therefore, increasing the abundance of the active compounds is critical for improving the quality of Isatidis Radix [7].

Polyploidy is widespread in plants, and nearly 70 % of angiosperms are polyploids including many important crops [8]. Polyploidization, also known as whole genome duplication (WGD), plays a pivotal role in promoting the evolution of plant morphological, physiological and reproductive diversity [8–12]. Compared with their diploid progenitors, polyploid plants often exhibit superior phenotypic traits, such as stronger tolerance, higher content of active compounds, and enlarged organs together with increased vigor [8, 13–16]. The most conspicuous features of polyploidy are the increased cell size, slowed cell division and tissue development, and increased organ size at maturity, which is referred to as the ‘gigas effect’ [11, 15]. The tetraploid *I. indigotica* accumulate more lignans than diploid, including lariciresinol and its derivatives, which present effective antiviral ingredients of *I. indigotica* [17]. The giant organs and enhanced concentrations of secondary metabolites realized by autopolyploidy are attractive for breeding the respective medicinal and agricultural plants.

However, there has been no report on the metabolomic and transcriptomic changes post polyploidization of Isatidis Radix until now. In the past several years, the research in the field of polyploidy is mainly focused on the transcriptional level, using RNAseq-based transcriptomic analysis to reveal the relationship between polyploidization and gene expression [16]. At present, only two reports of the Chinese Woad leaf (Isatidis Folium) transcriptomic changes induced by autotetraploidization were available [18, 19]. However, the root (Isatidis Radix) differed from leaf (Isatidis Folium) whether in biological function or in medicinal usage. And there are three monographs of *I. indigotica* included in the Chinese Pharmacopoeia, namely Isatidis Folium, Indigo Naturalis and Isatidis Radix [20], so these three kinds of Chinese herbal medicine preparations are somewhat different. Moreover, the metabolome is closer to phenotype than transcriptome.

Given that the metabolic activity was altered by the fluctuated gene expression, which led to the change of the concentration of secondary metabolites, we carried out a comparative transcriptomic and metabolomic study on Isatidis Radix autotetraploid and its progenitor. Through the integrative analysis of Isatidis Radix transcriptome and metabolome, the differentially expressed genes affecting the metabolic pathway of active components such as lignan were identified. As a result, the gene–lignan pathway network analysis highlighted polyploidy–responsive key functional genes and regulators.

## Materials and methods

### Plant materials and sampling

Appropriate permissions for collection and use of seed of *Isatis indigotica* Fort. (2*n* = 2*x* = 14) was obtained from Jiangsu Germplasm Repository Center. *I. indigotica* (2*n* = 2*x* = 14) used as diploid donor. Autotetraploid *I. indigotica* was artificially synthesized by colchicine-mediated polyploidy induction in vitro as described previously [16, 19]. Briefly, adventitious buds induced from diploid planet were subjected to 0.20 % colchicine treatment for 12 h, and transferred to MS medium without colchicine for 2 weeks. Then, the synthesized autotetraploid plantlets were transferred to 1/2 MS medium for rooting. The root tips (0.5-1 cm long) were excised and pretreated with 2 mmol·L^− 1^ 8-hydroxyquinoline solution for 4 h, and fixed with Carnoy’s solution at 4 °C for 24 h. Samples were then hydrolyzed using 1 mol·L^− 1^ HCl at 60 °C for 10 min. The hydrolyzed root tips were soaked in a drop of Carbol fuchsin for 10 min and squashed on the microscopic slide to observe the metaphase chromosomes. Finally, seedlings with roots were transplanted into nutritional soil. The diploid and autotetraploid *I. indigotica* seedlings were planted in the experimental fields in our campus for 1-year. Then, their roots were sampled for the subsequent transcriptomic and metabolomic analysis with three repeats (Fig.S1). The sampled fresh roots of *I. indigotica* were frozen with liquid nitrogen, transported and stored at -80 ℃.

### Targeted metabolomic analysis of Isatidis Radix metabolites

The Isatidis Radix samples were freeze-dried and ground into fine powder for metabonomic analysis. The widely targeted metabolic profile and quantitative detection of metabolites were performed by MetWare Biotechnology Co.,Ltd (Wuhan, China) (www.metware.cn). The quantification of metabolites was carried out using a predetermined multi-reaction monitoring method [21].

The elemental composition and mass fragmentation were compared to those registered inaccessible databases of NIST as well as the standards in a database compiled by MetWare Biotechnology Co.,Ltd [22].

### RNAseq libraries preparation and sequencing

Total RNA for RNAseq was extracted from seedling roots and about 1 µg RNA per sample was used as input material for the RNA sample preparations. Sequencing libraries were generated using NEBNext® UltraTM RNA Library Prep Kit for Illumina® (NEB, USA) and index codes were added to attribute sequences to each sample. For high-throughput sequencing, the library preparations were sequenced on an Illumina Hiseq X Ten platform and 150 bp paired-end reads were generated [19]. After the adaptor and low-quality sequences were trimmed, a total of 38.71 Gb clean data from 6 cDNA libraries were retained (Table S1).

### Mapping pair-end reads to the reference genome

The ‘Tuxedo’ package HISAT-StringTie [23] was utilized to process the RNAseq data. The reference genome (https://ndownloader.figshare.com/files/16341227) and gene model annotation file (https://ndownloader.figshare.com/files/16341245) were downloaded from Figshare [24].

The RNAseq reads for each sample were mapped to the reference genome using HISAT2, and the output SAM files were sorted and converted to BAM files using SAMtools (version 0.1.19). Then the sorted alignments were assembled into transcripts and the expression levels of all genes and transcripts were estimated using StringTie.

### Analysis of the differentially expressed genes (DEGs)

The expression values were represented by fragments per kilobase transcript per million reads mapped (FPKM), and the differential expression analysis of genes and transcripts across two conditions was performed using the Cuffdiff utility. Foldchange ≥ 2 and FDR ≤ 0.05 was set as the threshold to determine the DEGs between the compared samples. The KEGG pathway enrichment analysis of DEGs was conducted by Path_finder software with Q-value ≤ 0.1 [19].

### Integrative targeted metabolomic and transcriptomic profiling analysis

The data of metabolites profiling were normalized and exported to Simca-P software (12.0, http://www.umetrics.com/simca) employing partial least-squares discriminant analysis (PLS-DA) model. The differentially expressed metabolites were discriminated according to a threshold of variable importance in the projection (VIP) values (VIP > 1) after PLS-DA processing using the previously published protocols [17].

The correlation among lignan biosynthetic genes and lignans was constructed using the Pearson correlation coefficient according to the co-occurrence principle. The correlation network was generated using Cytoscape [17].

### qRT-PCR Analysis

In order to verify the differentially expressed genes (DEGs), the total RNA of 3 individuals of each genotype which were used for the aforementioned metabolite profiling was extracted by Total RNA Kit II (Qiagen). Then, DNaseI treatment, RNA concentration measurement and cDNA synthesis were carried out. According to the RNA-seq data, Primer5 software was used to design primer pairs for randomly selected DEGs (primers were listed in the supplementary Table S2). The housekeeping gene *UBIQUITIN1* was used as the reference gene to calculate the relative expression of genes using the comparative Ct method [11].

## Results

### Metabolomic alterations in Isatidis Radix following autopolyploidization

In order to assess the impact of polyploidization on the metabolomic shifts, the extracts from Isatidis Radix autotetraploids and their diploid parents were subjected to the targeted metabolic profiling by UPLC-TOF/MS. Totally, 283 annotated metabolites were identified in Isatidis Radix, the roots of *I. indigotica*.

**Fig. 1:**
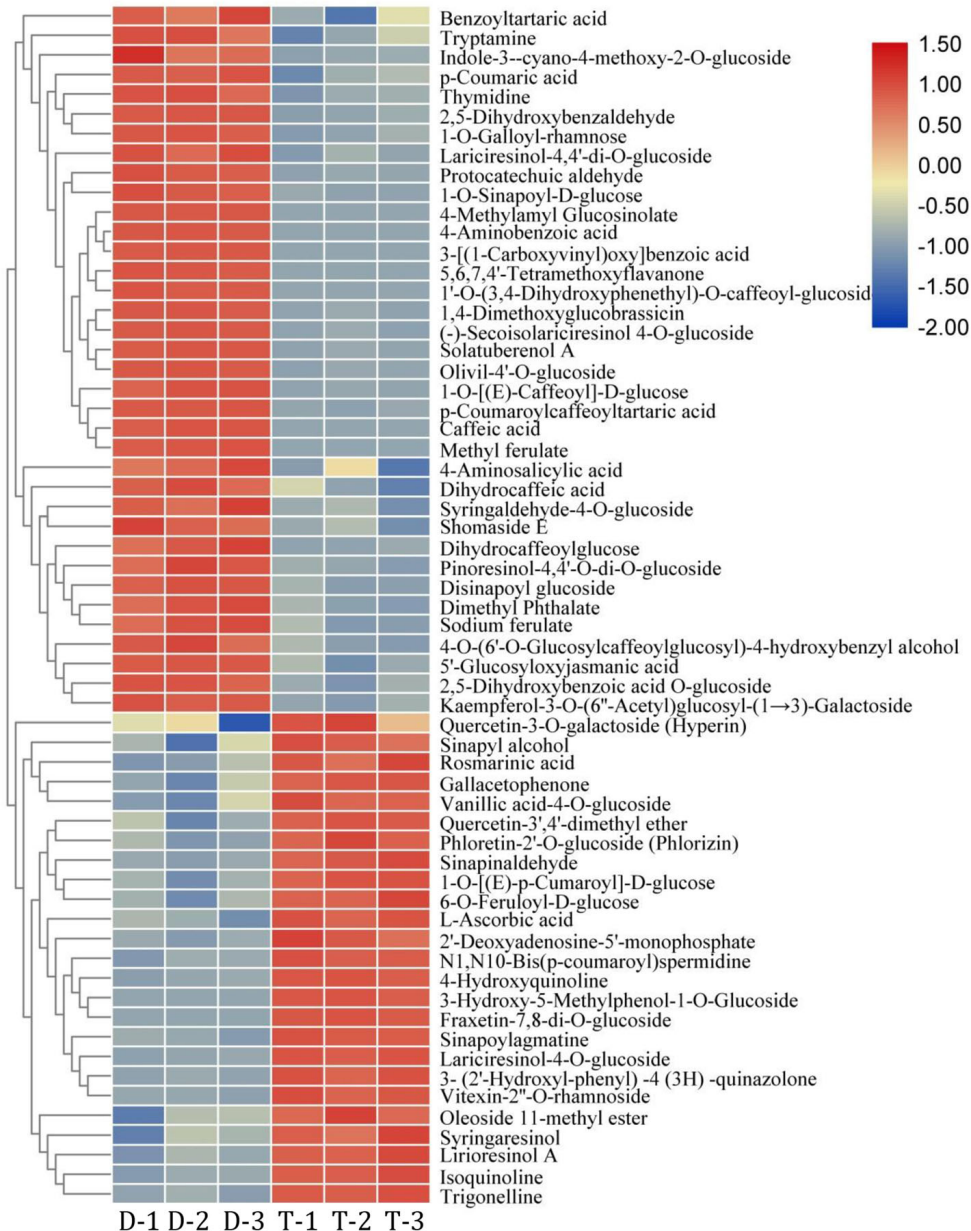
The polyploidization-altered metabolitesin Isatidis Radix. D and T with triplicate represents for Isatidis Radix diploid and tetraploid, respectively. Those polyploidization-altered metabolites (VIP > 1 and FC > 2) with no structural isomers were shown in the Heatmap

**Fig. 2:**
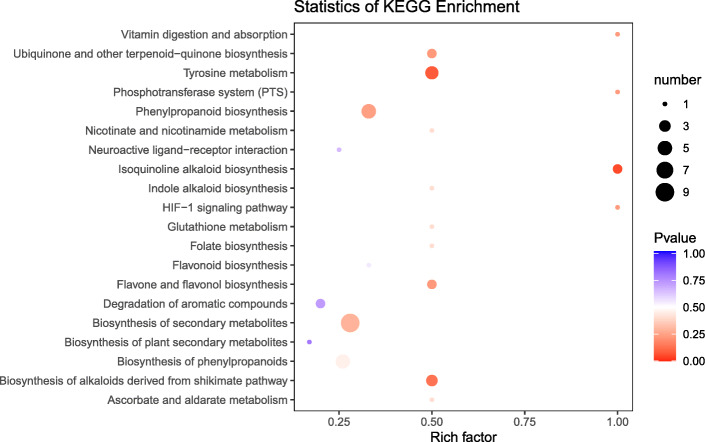
Polyploidy-inducibility of phenylpropanoids biosynthesis in Isatidis Radix

For the 70 polyploidization-altered metabolites (VIP > 1 and |log_2_FC|>1) (Fig. 1), they were mainly enriched in Alkaloids (including quinolines), Phenolic acids, Lignans, and Flavonols (Table S3). Given that lignans and flavonoids are the two major classes of phenylpropanoids in *I. indigotica*, the obtained results illustrated the polyploidy-inducibility of phenylpropanoids biosynthesis in Isatidis Radix obviously (Fig. 2).

**Fig. 3:**
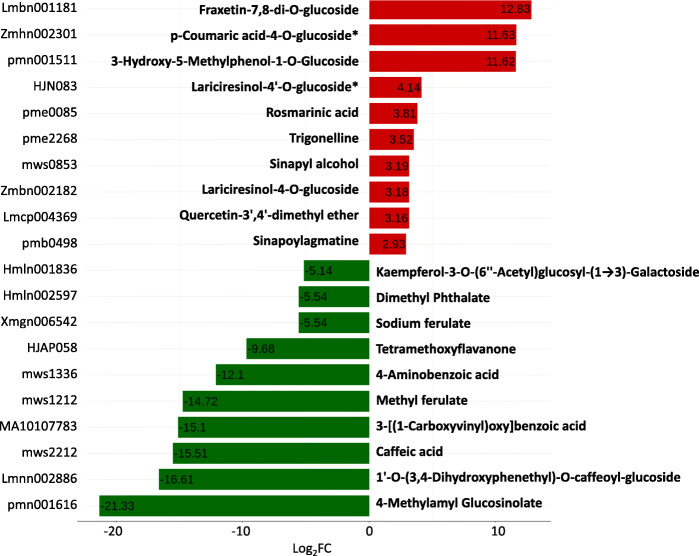
Top 20 altered metabolites in Isatidis Radix following autopolyploidization. The number in the colored band means log_2_(tetraploid/ diploid). The metabolites with the star signal * mean structural isomer detected

Compared with the diploid parent, the content of Fraxetin-7,8-di-O-glucoside was maintained at a higher level at autotetraploid (log_2_FC = 12.8), whereas 4-Methylamyl Glucosinolate was in the reverse trend (log_2_FC=-21.3). Next, p-Coumaric acid-4-O-glucoside (log_2_FC = 11.63) and 3-Hydroxy-5-Methylphenol-1-O-Glucoside (log_2_FC = 11.62) represented the second and the third largest upregulated metabolites following polyploidization, respectively, while 1’-O-(3,4-Dihydroxyphenethyl)-O-caffeoyl-glucoside (log_2_FC=-16.6) and Caffeic acid (log_2_FC=-15.5) was the second and the third significantly downregulated compounds by polyploidization, respectively (Fig. 3, Table S3).

It is noteworthy that the biosynthesis of Lariciresinol glucosides was enhanced in autopolyploid (Fig. 3, Table S3). Regarding the flavonoid, the accumulation of Phlorizin was enhanced in autotetraploid in comparison to diploid (Table S3). These compounds can be regarded as the indictor components of polyploidization based on metabolomics data.

### Polyploidization-modulated changes in lignan biosynthesis

Metabolic analysis revealed that the production of lignans was significantly increased post polyploidization, since Coniferyl alcohol and Lariciresinol glucosides accumulated more in autotetraploid than in diploid. Moreover, Coniferyl aldehyde and the subsequent Coniferyl alcohol, the critical precursor for lignan biosynthesis, were all enhanced in autotetraploid.

However, ferulic acid, the precursor of Coniferyl alcohol as well as Sinapyl alcohol, was less accumulated in autotetraploid than in diploid. Pinoresinol and its derivatives (Pinoresinol-4-O-glucoside, Pinoresinol-4,4’-O-di-O-glucoside) displayed less accumulation in autotetraploid than in diploid, as was the case for Secoisolariciresinol glucoside or Lariciresinol-4,4’-di-O-glucoside (also named clemastanin B) (Table S3), which implied that polyploidization-modulated changes in lignan biosynthesis.

### Polyploidization-responsive genes in Isatidis Radix

**Fig. 4:**
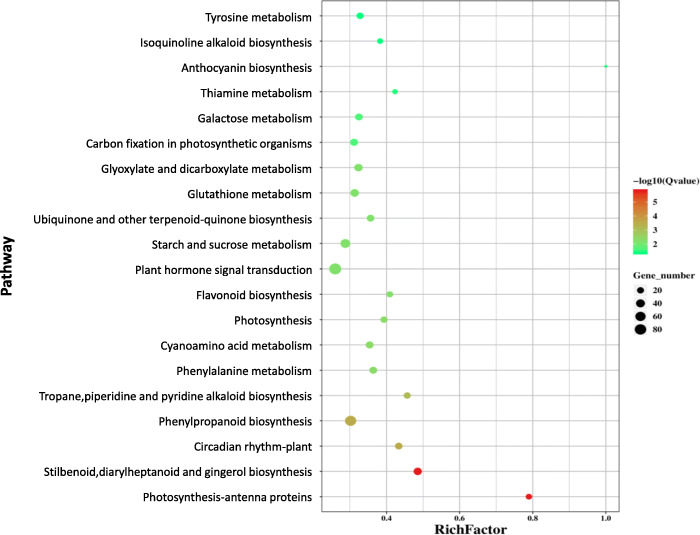
The KEGG pathway enrichment analysis of polyploidy-altered DEGs in Isatidis Radix

RNAseq-based transcriptomic profiling was performed to investigate the polyploidization imposed profound impacts on gene expression and the subsequent metabolic pathways in Isatidis Radix. Using a stringent cutoff (Foldchange > 2 and FDR ≤ 0.05), a total of 2065 differentially expressed genes (DEGs) were identified as being polyploidization-responsive genes, of which 1251 were polyploidy-induced and 814 were polyploidy-repressed in *I. indigotica* seedlings roots (Table S4). To further evaluate the functions and the biological pathways represented by the DEGs, we compared these genes with that included in the KEGG database [25]. The annotation and classification of root DEGs indicated that the polyploidization-altered root genes were enriched in phenylpropanoid biosynthesis and plant hormone signal transduction (Fig. 4).

To gain insights into the functionality of the 2065 DEGs that are likely to be associated with the process of polyploidization, all of these polyploidy-responsive transcripts were functionally grouped (Table 1). Among DEGs mainly involved in stress response, L-ascorbate peroxidase S, trehalose-phosphate synthase TPS7 and Senescence/dehydration-associated protein were upregulated. Regarding the upregulated DEGs involved in growth and development, Light-regulated protein 1, GIGANTEA, Glycine-rich protein 3 and two HAIKU members were of particular interest. In the kinase and signaling category, three kinase (Receptor-like protein kinase FERONIA, Wall-associated receptor kinase WAK14 and Mitogen-activated protein kinase MPK19) and Gibberellin receptor GID1C were upregulated by polyploidization. Regarding the transporters, Glutathione S-transferase GSTZ1, Sulfate transporter AST12 and two ABC transporter C family members (ABCG14/36) were upregulated by polyploidization (Table 1).

**Table 1:** Functional classification of some differentially expressed genes (DEGs) in Isatidis Radix

Polyploidy-upregulated			Polyploidy-downregulated		
**Gene ID**	**Log**_**2**_**FC**	**Annotation**	**Gene ID**	**Log**_**2**_**FC**	**Annotation**
**Stress**					
Iin06055	2.2	Temperature-induced lipocalin-1	Iin16550	-1.7	Universal stress protein A-like protein
Iin07195	6.1	L-ascorbate peroxidase S, chloroplastic/mitochondrial	Iin20218	-1.6	Thioredoxin H2
Iin08766	1.2	Trehalose-phosphate synthase TPS7	Iin26513	-1.2	Gamma carbonic anhydrase CAL2, mitochondrial
Iin11872	3.9	Cysteine proteinase RD21A	Iin28989	-2.2	Polyadenylate-binding protein PABP-2
Iin26982	1.5	Bifunctional enolase 2/transcriptional activator	Iin02876	-3.6	Jacalin-related lectin 35
Iin27472	1.8	Galactinol–sucrose galactosyltransferase 2	Iin24588	-1.2	Glutathione S-transferase DHAR2
Iin21449	1.2	Senescence/dehydration-associated protein, chloroplastic			
Iin15549	3.8	Universal stress protein A-like protein			
**Growth and development**					
Iin01550	5.7	Light-regulated protein 1, chloroplastic	Iin18645	-1.6	Arogenate dehydratase PDT1, chloroplastic
Iin10290	2.2	Protein GIGANTEA	Iin05822	-1.3	Adenylate kinase isoenzyme 6 homolog
Iin15495	2.5	Mediator of RNA polymerase II transcription subunit 37c	Iin10029	-3.7	Auxin-responsive protein SAUR50
Iin02027	1.7	HAIKU1/VQ14	Iin11047	-1.7	Rop guanine nucleotide exchange factor 14
Iin13953	3.2	Glycine-rich protein 3	Iin12932	-3.2	Profilin-1
Iin00462	2.9	BTB/POZ and TAZ domain-containing protein 1	Iin23516	-1.2	Rac-like GTP-binding protein ARAC3
Iin04656	3.7	MADS-box protein FLOWERING LOCUS C	Iin00261	-1.2	Agamous-like MADS-box protein AGL31
Iin05649	2.0	Nucleolin 1	Iin07151	-2.7	DNA (cytosine-5)-methyltransferase 1
Iin08418	4.8	E3 ubiquitin ligase SUD1	Iin12525	-11.7	Uracil phosphoribosyltransferase, chloroplastic
Iin09123	1.8	Receptor-like protein kinase HAIKU2	Iin17933	-1.1	Pyruvate dehydrogenase E1 component subunit beta-3, chloroplastic
Iin09717	2.5	Glyceraldehyde-3-phosphate dehydrogenase GAPCP2, chloroplastic	Iin19209	-1.3	UDP-sugar pyrophosphorylase
Iin15883	1.5	Phosphoinositide PLC2	Iin25514	-1.6	Zinc finger protein 6
			Iin27501	-1.3	SUMO-conjugating enzyme SCE1
			Iin29572	-1.9	Adenosylhomocysteinase 1
**Kinase and signaling**					
Iin26824	1.2	Receptor-like protein kinase FERONIA	Iin17733	-2.2	LRR receptor-like protein kinase PXC1
Iin19551	1.7	Wall-associated receptor kinase, WAK14	Iin01347	-1.1	Histidine-containing phosphotransfer protein 2
Iin24110	6.0	Mitogen-activated protein kinase, MPK19	Iin03883	-1.6	Phosphoglucan, water dikinase, chloroplastic
Iin27283	1.2	receptor-like protein kinase	Iin10356	-7.3	Hexokinase-like 1 protein
Iin29680	4.3	LRR receptor-like kinase	Iin28764/ Iin30061	-1.6	RPM1-interacting protein 4
Iin01264	1.0	LRR receptor-like kinase	Iin22433	-5.3	F-box protein GID2
Iin00785	2.2	Remorin	Iin29325	-2.2	Auxin-responsive protein IAA7
Iin01090	1.8	Gibberellin receptor GID1C	Iin05103	-1.7	Gibberellin-regulated protein 4
Iin07657	3.1	Copper amine oxidase	Iin06703/ Iin21095	-1.1	Small acidic protein 1
**Transporter**					
Iin01771	2.7	Glutathione S-transferase, GSTZ1	Iin22173	-1.7	Calcium permeable stress-gated cation channel, CSC1
Iin04670	1.6	Ras-related protein, RABH1e	Iin11860	-1.3	ABC transporter A family, ABCA4
Iin26854	2.3	Sulfate transporter, AST12	Iin04029	-1.3	Membrane magnesium transporter
Iin10902	1.8	CSC1-like protein, ERD4	Iin28450	-0.8	Mitochondrial phosphate carrier protein, MPT3
Iin01411	2.2	AAA-ATPase	Iin22985	-5.9	Venom phosphodiesterase
Iin27921/ Iin23995	1.6	ABC transporter C family, ABCG14/36			
Iin23984	1.1	Amino acid transporter, AVT3C			

Log_2_FC means log_2_(FPKM-tetraploid/FPKM-diploid). FPKM means the fragments per kilobase transcript per million reads mapped by RNAseq analysis

Furthermore, 26 polyploidy-altered transcriptional factors (FPKM > 10 in one sample) were identified in Isatidis Radix (Table 2). Among them, 18 transcriptional factors TFs (including bZIP40, NAC29, Myb59) and 8 TFs (including ERF36/70 and NAC41) were up-regulated and down-regulated by polyploidization, respectively.

**Table 2:** Polyploidy-altered transcriptional factors in Isatidis Radix

Name	Diploid	Tetraploid	Log_2_FC	ID	Chromosome	Start	End	Strand	Annotation
ARF14	7.2	62.6	3.2	Iin01634	Lachesis_group0	8,536,701	8,537,541	-	AP2/ERF and B3 domain
RAV2	2.1	13.4	2.8	Iin25260	Lachesis_group6	7,129,978	7,131,110	-	AP2/ERF and B3 domain
bZIP40	29.1	61.9	1.1	Iin02491	Lachesis_group0	15,255,544	15,256,922	+	bZIP transcription factor
CDC5	8.7	24.3	1.5	Iin13523	Lachesis_group3	7,021,761	7,028,721	-	Cell division cycle 5-like
ERF36	90.0	19.1	-2.2	Iin15129	Lachesis_group3	27,555,240	27,555,962	-	Ethylene-responsive factor
ERF55	3.2	16.5	2.4	Iin11510	Lachesis_group2	20,968,841	20,969,965	-	Ethylene-responsive factor
ERF70	166.1	56.9	-1.5	Iin24996	Lachesis_group6	5,265,430	5,265,968	-	Ethylene-responsive factor
HsfA1a	10.9	2.4	-2.2	Iin10321	Lachesis_group2	10,028,288	10,028,973	+	Heat stress transcription factor
HsfB2b	4.8	10.2	1.1	Iin07593	Lachesis_group1	30,892,038	30,894,605	+	Heat stress transcription factor
REV	3.5	22.5	2.8	Iin02879	Lachesis_group0	24,168,399	24,168,936	+	Homeobox-leucine zipper
NAC41	76.6	8.8	-3.2	Iin18045	Lachesis_group4	8,178,123	8,179,515	-	NAC domain-containing
NAC29	1.9	169.4	6.5	Iin25189	Lachesis_group6	6,708,153	6,709,151	-	NAC domain-containing
NAC47	4.5	16.4	1.9	Iin16287	Lachesis_group3	33,189,224	33,191,152	+	NAC domain-containing
NAC54	5.7	21.9	2.0	Iin19433	Lachesis_group4	29,656,612	29,657,225	+	NAC domain-containing
NF-YC-1	16.9	4.2	-2.0	Iin08913	Lachesis_group2	2,970,892	2,972,650	-	Nuclear transcription factor
bHLH44	11.2	0.1	-6.9	Iin09913	Lachesis_group2	7,785,960	7,787,812	+	Basic helix-loop-helix protein
bHLH129	5.4	14.0	1.4	Iin17039	Lachesis_group4	2,495,161	2,499,719	+	Basic helix-loop-helix protein
bHLH130	2.0	23.3	3.6	Iin17124	Lachesis_group4	2,983,700	2,986,470	+	Basic helix-loop-helix protein
bHLH63	7.0	14.7	1.1	Iin23473	Lachesis_group5	35,187,500	35,189,238	+	Basic helix-loop-helix protein
GTE4	21.1	76.8	1.9	Iin08753	Lachesis_group2	2,302,922	2,306,873	+	Transcription factor GTE4
Myb51	6.5	31.6	2.3	Iin09927	Lachesis_group2	7,849,190	7,851,367	+	MYB Transcription factor
Myb59	19.5	317.8	4.0	Iin05872	Lachesis_group1	10,463,654	10,464,989	+	MYB Transcription factor
ASIL2	13.6	29.5	1.2	Iin14214	Lachesis_group3	21,584,634	21,585,940	-	Trihelix transcription factor
ASR3	14.7	3.5	-2.0	Iin23152	Lachesis_group5	33,490,036	33,491,219	-	Arabidopsis SH4-Related3
WRKY16	4.3	18.5	2.2	Iin21936	Lachesis_group5	26,229,642	26,232,137	+	WRKY transcription factor
WRKY19	13.1	0.8	-3.9	Iin00005	Lachesis_group0	36,353	38,261	-	WRKY transcription factor

The number in the column of Diploid and Tetraploid is the FPKM (fragments per kilobase transcript per million reads mapped) value by RNAseq analysis, and log_2_FC means log_2_(FPKM-tetraploid/FPKM-diploid)

### Systematic transcriptomic and metabolomic shift post polyploidization

To integrate the analysis of polyploidy-responsive metabolome and transcriptome, a canonical correlation analysis using Pearson’s correlation coefficient was performed to display the dynamic variation over the polyploidization course. This integrative analysis showed that 1584 DEGs were highly correlated with the 70 polyploidization-altered metabolites, with |PCC|>0.917 (Table S5).

**Fig. 5:**
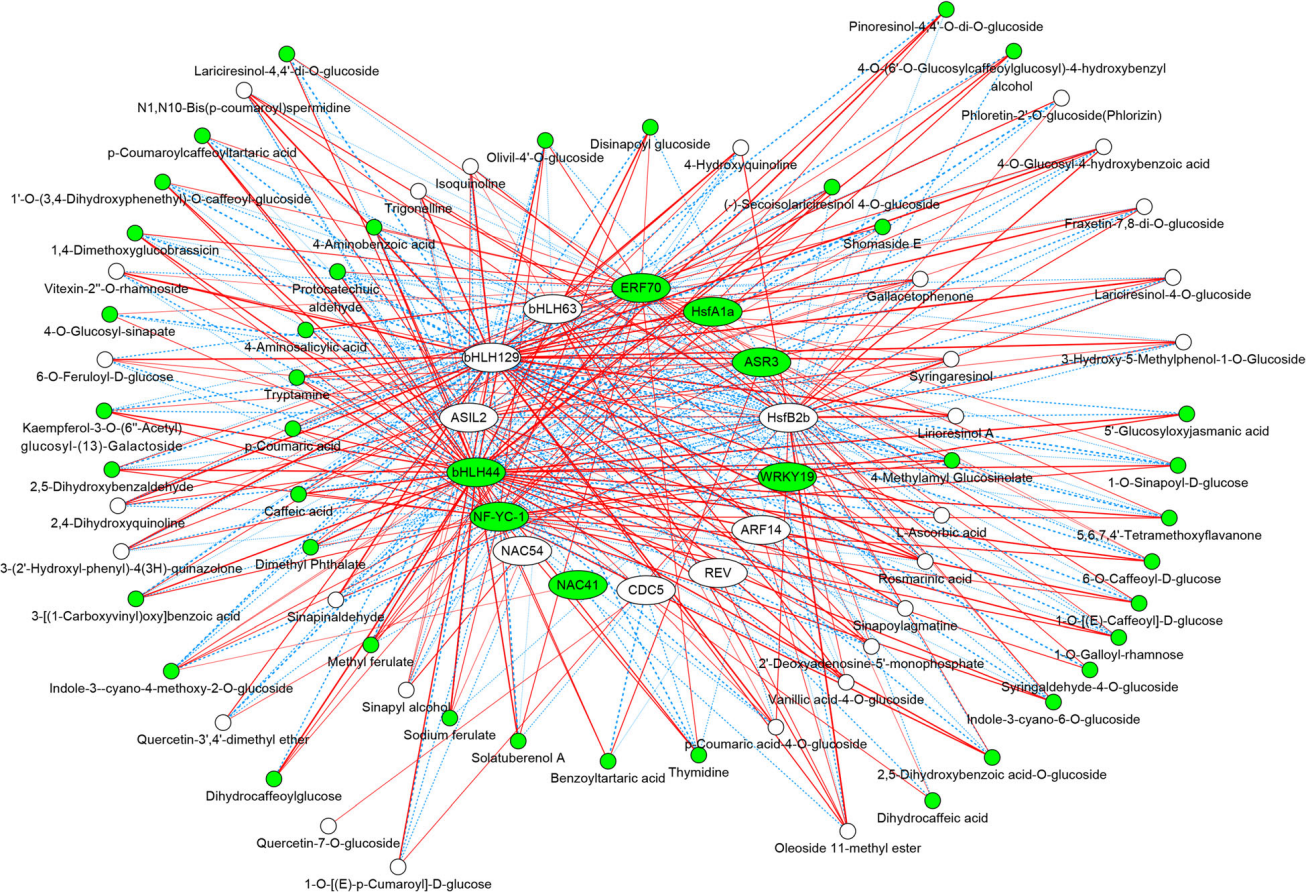
The network of polyploidy-altered transcriptional factors and metabolites in Isatidis Radix

A TF-metabolite correlation network was built that consisted of 15 polyploidy-altered TFs and 67 fluctuated compounds to characterize TFs involved in polyploidy-induced alteration in roots metabolome and transcriptome (Fig. 5, Table S6).

The polyploidy-downregulated metabolites or transcriptional factor genes are marked with background in green circle or oval, respectively. Red lines indicate the positive correlations while blue lines indicate the negative correlation. The polyploidy-altered pattern and annotation of metabolites or genes are given in Table S6.

Among the 15 polyploidy-altered TFs, bHLH44/63/129, ERF70, ASIL2, NF-YC-1 and HsfB2b were highly connected to the fluctuated compounds. It is also intriguing to note that the polyploidy-induced NAC54 was positively correlated with polyploidization-enhanced two metabolites (Sinapyl alcohol and Quercetin-3’,4’-dimethyl ether).

For the polyploidization-enhanced metabolites, three types of Quinolines, Gallacetophenone, Sinapyl alcohol, *p*-Coumaric acid-4-o-glucoside, Vanillic acid-4-o-glucoside, Rosmarinic acid, Lirioresinol A, Syringaresinol, Lariciresinol-4-o-glucoside, Fraxetin-7,8-di-o-glucoside, Vitexin-2’’-o-rhamnoside were all positively correlated with 4 polyploidy-induced TFs (HsfB2b, ASIL2, bHLH129, bHLH63).

However, the aforementioned metabolites together with Phloretin-2’-O-glucoside (Phlorizin) and Quercetin-3’,4’-dimethyl ether were all negatively correlated with 3 polyploidy-suppressed TFs (NF-YC-1, bHLH44 and ERF70) except Syringaresinol negatively with NAC41. Moreover, bHLH129 was positively correlated with L-Ascorbic acid specifically, while it was connected to Phloretin-2’-O-glucoside (Phlorizin) and Quercetin-3’,4’-dimethyl ether together with other TFs. bHLH44 and ERF70 aforementioned were all negatively correlated with L-Ascorbic acid (Fig. 5, Table S6).

### Integrated metabolomic and transcriptomic analysis of lignan metabolism modulated by polyploidization

To have a systematic view on the polyploidy-responsive variation of lignan biosynthesis, the transcripts involved in the general phenylpropanoid pathway, lignan biosynthesis and the corresponding metabolites were subjected to construct lignan biosynthesis pathway.

**Fig. 6:**
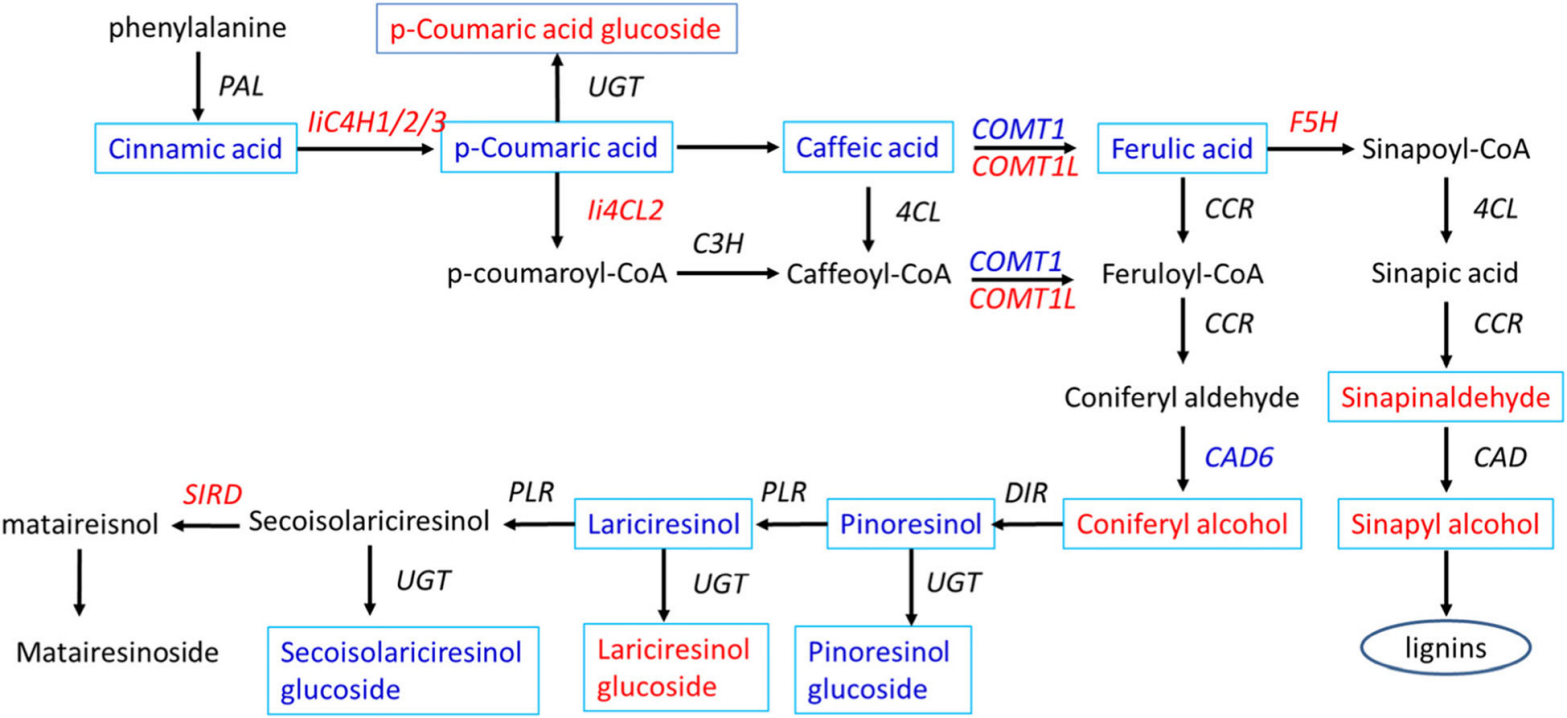
The lignan metabolism was modulated by polyploidization in Isatidis Radix

Several key metabolites, involved in general phenylpropanoid pathway (e.g. Coniferyl aldehyde and Coniferyl alcohol) [25] and lignan compound (e.g. Lariciresinol glucoside), were markedly increased post polyploidization. Moreover, various catalytic genes (e.g. C4H, 4CL, COMT and F5H) showed similar up-regulated patterns in correspondence with the increased metabolites (Fig. 6, Table S3, Table S4), suggesting lignan biosynthesis pathway modulated by polyploidization with transcriptomic and metabolomics evidence.

PAL, phenylalanine ammonia-lyase; UGT, UDP-sugar-dependent glycosyltransferase; C3H, *p*-coumarate 3-hydroxylase, 4CL, 4-(hydroxy) cinnamoyl CoA ligase; CCR, cinnamoyl‐CoA reductase; F5H, ferulate‐5‐hydroxylase; CAD, cinnamyl alcohol dehydrogenase; DIR, dirgent protein; PLR, pinoresinol/lariciresinol reductase; SIRD, secoisolariciresinol dehydrogenase. Metabolites or catalytic genes up-regulated post polyploidization were marked in red, and those down-regulated post polyploidization were in blue. The polyploidy-altered metabolites were shown in box, while the polyploidy-altered genes were placed adjacent to the arrows. Metabolites or genes in black means not significantly regulated by polyploidization, and the polyploidy-altered pattern and annotation information of metabolites or genes are given in supplementary Table S3 and Table S4, respectively.

**Fig. 7:**
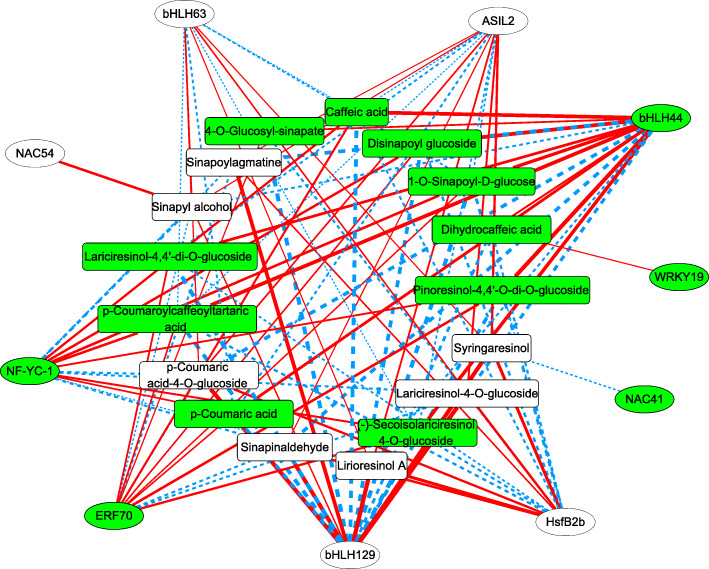
The network of polyploidy-altered transcriptional factors and lignans in Isatidis Radix

In the polyploidy-altered TFs-lignans network, there were 10 polyploidy-altered TFs and 17 fluctuated phenylpropanoid pathway compounds, which indicated the transcriptomic and metabolic shifts in lignan metabolism as a result of polyploidization-mediated transcriptional regulation (Fig. 7, Table S7).

Among the 10 polyploidy-altered transcriptional factors, polyploidy-inhibited bHLH44 and polyploidy-induced bHLH129 were highly correlated with all the fluctuated phenylpropanoid pathway compounds (7 up and 10 down) except Syringaresinol, but with the reverse trend.

The polyploidization-enhanced Lariciresinol-4-O-glucoside and polyploidization-reduced (-)-Secoisolariciresinol 4-O-glucoside were correlated with 4 polyploidy-induced TFs (HsfB2b, ASIL2, bHLH129, bHLH63) and 3 polyploidy-suppressed TFs (NF-YC-1, bHLH44 and ERF70) with reverse trend, so was the case for the polyploidization-reduced Lariciresinol-4,4’-di-O-glucoside correlated with the aforementioned TFs except bHLH63 or NF-YC-1 (Fig. 7, Table S7).

The polyploidy-downregulated metabolites or genes are marked with background in green box or oval, respectively. Red lines indicate the positive correlations while blue lines indicate the negative correlation. The polyploidy-altered pattern and annotation of metabolites or genes are given in supplementary Table S7.

## Discussion

### Polyploidization contributed to the ‘gigas effect’ and high content of active compounds in ***I. indigotica*** autotetraploid roots

Polyploidization, also known as whole-genome duplication (WGD), results in the “gigas effect” that includes increased cell size, enlarged vegetative or reproductive organs and prolonged vegetative growth [11, 15, 26]. Compared to their diploid progenitors, the autotetraploid *I. indigotica* plants exhibit bigger robustness and larger leaves with deeper color, which was in accordance with the “gigas effect”.

Among DEGs mainly involved in stress response, L-ascorbate peroxidase S, trehalose-phosphate synthase (TPS7) and senescence/dehydration-associated protein were upregulated by polyploidization (Table 1). In Arabidopsis, TPS1 catalyzes the synthesis of the sucrose-signaling metabolite trehalose 6-phosphate which acts as a potent regulator of post-embryonic growth and development [27]. Moreover, rice OsTPS1 may improve the abiotic stress tolerance by increasing the accumulation of trehalose and proline, and modulating the expression of stress-related genes [28]. Regarding the senescence/dehydration-associated protein, Arabidopsis ERD7 and its homologs play essential roles in plant stress responses and development and are associated with modification of membrane lipid composition [29]. Therefore, the role of these polyploidy-enhanced genes in ‘gigas effect’ and stress tolerance of autotetraploid needs further establishment.

Regarding the kinase genes upregulated by polyploidization, the receptor-like protein kinase FERONIA was of particular interest (Table 1). In Arabidopsis, the couple of extracellular peptide RAPID ALKALINIZATION FACTOR1 (RALF1) and FERONIA (FER) acted as a central hub between the cell surface and downstream signaling events, and the RALF–FER pathway functioned as an essential regulator of plant stress responses [30]. Furthermore, the RALF1-FER-GRP7 module provided a paradigm for regulatory mechanisms of RNA splicing to regulate plant fitness and flowering time [30, 31]. Glycine-rich proteins (GRPs) were demonstrated to participate in cold stress responses, plant defense, cell elongation and fertility. Moreover, rice glycine-rich protein *OsDG2* plays important roles in chloroplast development during early seedling stage [32]. In this study, glycine-rich protein (*Iin13953*) was one of the polyploidy-upregulated DEGs which were involved in growth and development (Table 1). Hence, it is interesting to investigate the contribution of polyploidy-induced FER together with glycine-rich protein *Iin13953* to the modulation of cell growth and stress responses.

Arabidopsis root hair defective 6-like 4 (RSL4), a bHLH transcription factor, triggers the expression of hundreds of root hair genes which promote ectopic root hair growth, and the autocrine regulation of root hair size by the RALF-FERONIA-RSL4 signaling pathway has been revealed [33]. In this study, polyploidy-induced bHLH129 and bHLH63 together with FER were identified (Tables 1 and 2), but whether they acted as central hubs orchestrating complex intracellular and extracellular signals required further elucidation.

One of the ideal expectations for the medicinal autopolyploid was that the organ giantism was accompanied by the higher content of some chemical compositions, especially the active compounds [8, 13–16, 19]. *I. indigotica*, like *I. tinctoria* of the *Brassicaceae* family, represents a valuable source of bioactive compounds such as alkaloids, phenolic compounds, phenylpropanoids and terpenoids [3, 19]. In this study, the content of active compounds in the roots of autotetraploid *I. indigotica* was higher than that in diploid roots, and some new compounds including Phlorizin, Tannins and Solatuberenol A were also isolated in Isatidis Radix (Table S3). Moreover, Isatidis Radix autotetraploid accumulated more Lariciresinol glucosides than the diploid counterparts, consistent with previous report [17]. Known indolic alkaloids called Indirubin and indicant (iso) which were reported in the dried *I. tinctoria* leaves [2, 3] were also identified to be polyploidy-upregulated metabolite in this study (Table S3). These collectively implied the potentiality of enhancing active compounds accumulation through polyploidization.

The penetration-resistance gene PEN3/ABCG36/PDR8 and PDR12 function redundantly to mediate the secretion of camalexin, and they have multiple functions in Arabidopsis immunity via transport of distinct Trp metabolic products [34]. *PEN2* encodes a myrosinase that catalyzes the degradation of indole glucosinolates, and the catalyzed products of PEN2 are postulated to be transported to the apoplast by PEN3. Moreover, the indole compound 4-methoxyindole-3-methanol that is a substrate for PEN3 stimulates bacterial flg22-induced callose deposition [35]. In this study, one myrosinase (*Iin26136*) and two myrosinase-binding protein genes were induced by polyploidization (Table S4). Nonetheless, whether the coordinated function mechanism of PEN2-PEN3 play its role in synthesis and export of the active metabolites (including but not limited to indole compounds) in Isatidis Radix merits further investigation.

### Gene–lignan pathway network analysis highlighted polyploidy–responsive key functional genes and regulators

Phenylpropanoid is the major group of secondary metabolites, which metabolism generate diverse metabolites including lignans and flavonoids, and lignans are identified to be the pharmacologically active compounds [7], therefore the correlations between the identified DEGs and phenylpropanoid pathway compounds were inferred based on the co-occurrence principle between the transcript and metabolite levels. Several key metabolites involved in general phenylpropanoid pathway (e.g. coniferyl aldehyde and coniferyl alcohol) and lignan compound (e.g. Lariciresinol glucoside) were markedly increased post polyploidization. Moreover, various catalytic genes (e.g. C4H, 4CL, COMT and F5H) showed similar up-regulated patterns in correspondence with the increased metabolites (Fig. 6), suggesting that transcriptomic and metabolomic profile of lignan biosynthesis pathway was modulated by polyploidization.

Transcriptional factors were predicted to act as key regulators of lignan synthesis in *I. indigotica.* Among the 10 polyploidy-altered TFs, polyploidy-inhibited bHLH44 and polyploidy-induced bHLH129 were highly correlated with all the fluctuated phenylpropanoid pathway compounds (7 up and 10 down) except Syringaresinol, but with the reverse trend (Fig. 7). It was also intriguing to note that the polyploidy-induced NAC54 was positively correlated with polyploidization-enhanced Sinapyl alcohol (Fig. 7). Further studies of the regulatory mechanism of polyploidy-induced bHLH129 and NAC54 may provide fruitful means to reveal the beneath mechanism for polyploidy vigor and lignan biosynthesis of *I. indigotica*.

It was reported that IiWRKY34 significantly contributed to the polyploidy vigor of *I. indigotica*, and IiWRKY34 positively contributed to the yield, lignan biosynthesis and stress tolerance in *I indigotica* hairy roots, however, this key regulator was not identified here using the genuine roots namely Isatidis Radix. One possible explanation is that the expression pattern of genes in the induced hairy roots of tetraploid *I. indigotica* greatly differed from that in its original root [17].

Not surprisingly, different TFs may play distinct roles in lignan biosynthesis and allow the autotetraploid roots to prioritize toward a more efficient lignan biosynthesis. Therefore, whether these highlighted TFs regulate DEGs for lignan biosynthesis is the most important issue to elucidate the genuine regulators of lignan biosynthesis in Isatidis Radix.

## Supplementary Information

**Additional file 1: Table S1. **Quality statistics of the filtered RNAseq reads.

**Additional file 2: Table S2.**qRT-PCR primers of Isatidis Radix genes.

**Additional file 3:Table S3. **Metabolomic profiling of Isatidis Radix.

**Additional file 4: Table S4.** Polyploidy-responsive genes in Isatidis Radix.

**Additional file 5: Table S5. **Correlation analysis of polyploidy-responsive genes and metabolites in Isatidis Radix.

**Additional file 6: Table S6. **Correlation analysis of transcriptional factors and metabolites in Isatidis Radix.

**Additional file 7: Table S7. **I. indigotica transcriptional factors acted as key regulators of lignan synthesis in Isatidis Radix.

**Additional file 8: Figure S1.** The morphological characterization of I. indigotica autotetraploid seedling and its diploid progenitor. A The *I. indigotica* seedling of autotetraploid (4x) and its diploid (2x). B Chromosomes of *I. indigotica* autotetraploid and diploid root tips. C The comparison of stomata between autotetraploid and diploid leaf. D Isatidis Radix autotetraploid and diploid. 

## Data Availability

The datasets supporting the conclusions of this article are included within the article and its additional files.
