# Supplementary figures and images for: Integrative analyses of targeted metabolome and transcriptome of Isatidis Radix autotetraploids highlighted key polyploidization-responsive regulators
Source: BMC Genomics. 2021 Sep 17;22:670. doi: 10.1186/s12864-021-07980-w (PMC8449450; doi:10.1186/s12864-021-07980-w)

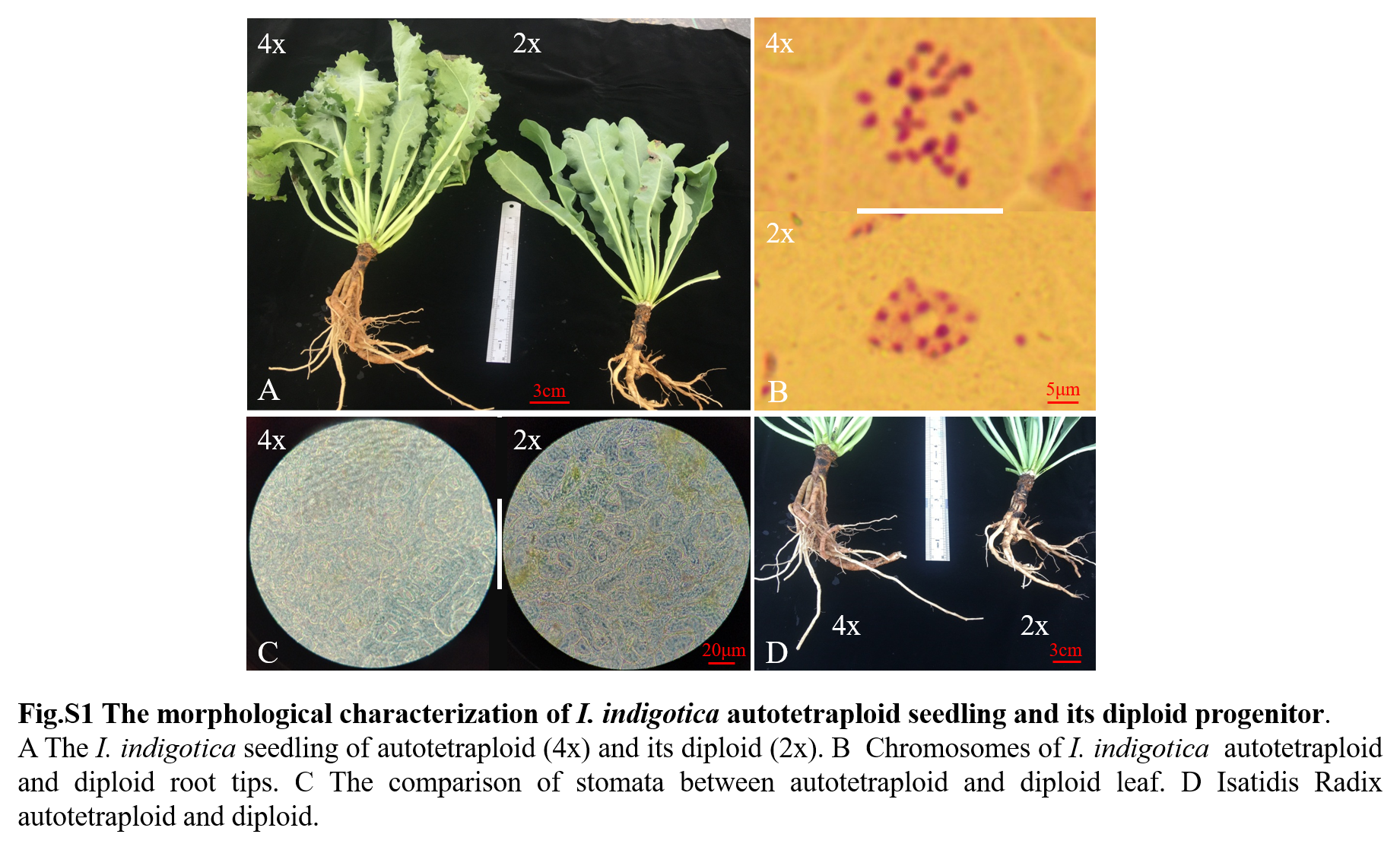

Supplement: Supplementary file 8 — Additional file 8: Figure S1. The morphological characterization of I. indigotica autotetraploid seedling and its diploid progenitor. A The I. indigotica seedling of autotetraploid (4x) and its diploid (2x). B Chromosomes of I. indigotica autotetraploid and diploid root tips. C The comparison of stomata between autotetraploid and diploid leaf. D Isatidis Radix autotetraploid and diploid. [file 12864_2021_7980_MOESM8_ESM.png]
